# Myelodysplastic syndrome in a 30-year-old man with coronavirus disease 2019 (COVID-19): a diagnostic challenge

**DOI:** 10.4322/acr.2021.274

**Published:** 2021-04-23

**Authors:** Xin Qing, Jennifer Cai, Adam Rock

**Affiliations:** 1 Harbor-UCLA Medical Center, Department of Pathology, Torrance, CA, USA; 2 Harbor-UCLA Medical Center, Department of Internal Medicine, Torrance, CA, USA

**Keywords:** Myelodysplastic Syndromes, COVID-19, Bone Marrow, Cytogenetics, SARS-CoV-2

## Abstract

**Background:**

Myelodysplastic syndromes (MDS) mainly occur in the elderly but can rarely affect younger individuals too. The correct diagnosis relies on careful morphologic evaluation, cytogenetic/molecular results, and excluding reactive conditions mimicking MDS. We present the clinical, pathologic, cytogenetic, and molecular features of a case of MDS with excess blasts-2 (MDS-EB-2) in a 30-year-old male who was found to have pancytopenia during his hospitalization for coronavirus disease 2019 (COVID-19) and discuss the diagnostic challenges of MDS in patients with COVID-19.

**Case presentation:**

A 30-year-old man presented to an outside hospital with fever, chills, weakness, coughing spells, dizziness and shortness of breath and was diagnosed with bilateral pneumonia due to COVID-19. At the outside hospital, he was found to be pancytopenic, and a subsequent bone marrow aspiration and biopsy raised concern for a COVID-19 induced hemophagocytic lymphohistiocytosis. In addition, MDS could not be ruled out. The patient was thus referred to our institute for further management. The patient’s peripheral blood showed pancytopenia with occasional dysplastic neutrophils and a few teardrop cells. Given the diagnostic uncertainty, a bone marrow aspiration and a biopsy were repeated revealing a hypercellular bone marrow with erythroid hyperplasia, megakaryocytic hyperplasia, trilineage dysplasia, increased blasts (13%), many ring sideroblasts, and mild to moderate myelofibrosis, consistent with MDS-EB-2. Chromosomal analysis revealed isochromosome 14. Next generation sequencing demonstrated SF3B1 K700E mutation.

**Discussion and conclusion:**

The diagnosis of MDS can be challenging, particularly in young patients. Cytopenia and myelodysplastic features have been reported in COVID-19 patients, making the diagnosis of MDS more elusive. A careful pathologic examination of the bone marrow with ancillary studies including flow cytometry, immunohistochemistry, and cytogenetic and molecular studies in combination with a thorough clinical evaluation, leads to the accurate diagnosis.

## INTRODUCTION

Myelodysplastic syndromes (MDS) are a heterogeneous group of clonal hematological conditions affecting the hematopoietic stem cells in the bone marrow, associated with ineffective hematopoiesis, and manifesting as peripheral cytopenias and morphologic dysplasia in hematopoietic elements with less than 20% blasts in blood or bone marrow. They are also characterized by recurrent genetic abnormalities and an increased risk of developing acute myeloid leukemia (AML). The bone marrow is often hypercellular. MDS primarily affect elderly patients. According to the Surveillance, Epidemiology and End Results (SEER) database, only about 3-6% of MDS patients are less than 50 years old at the time of diagnosis.^1^ ^,^ ^2^ There are several subtypes of MDS, including MDS with single lineage dysplasia (MDS-SLD), MDS with multilineage dysplasia (MDS-MLD), MDS with ring sideroblasts (MDS-RS), MDS with isolated del(5q) (MDS/del5q), MDS with excess blasts (MDS-EB), MDS, unclassifiable (MDS-U), and refractory cytopenia of childhood (provisional diagnosis).^3^ This classification considers blood and bone marrow blast proportion, the number of myeloid lineages exhibiting significant dysplastic changes (i.e., at least 10% of cells in a lineage demonstrate dysplastic features morphologically), the presence of ring sideroblasts or Auer rods, and, to a limited extent, karyotype [del(5q)] and molecular genetic finding (SF3B1 mutation). Accurate diagnosis and classification of MDS relies on careful evaluation of high-quality peripheral blood and bone marrow aspirate smears, adequate bone marrow biopsies, karyotype and molecular genetic results, as well as clinical information.

Here we describe a unique case of MDS-EB2 in a 30-year-old male patient with coronavirus disease 2019 (COVID-19), in whom the diagnosis was a challenge.

## CASE REPORT

A previously healthy 30-year-old man presented to an outside hospital with fever, chills, weakness, coughing spells, dizziness and shortness of breath. His chest X-ray showed bilateral lung with multifocal peripheral consolidation, highly suspicious for COVID-19 pneumonia. Subsequent testing confirmed SARS-CoV-2, and he was diagnosed with bilateral pneumonia due to COVID-19. His blood culture was reported as no growth. Vital signs remained stable and he did not require any supplemental oxygen. However, he was found to be pancytopenic, with macrocytic anemia and an inappropriately low reticulocyte count. He was also found to have hyperferritinemia (ferritin level >100,000 ng/mL). His vitamin B12, iron, folate, haptoglobin levels were all within reference ranges, with no evidence of hemolysis. He had quit drinking alcohol 2 years ago and had no recent illicit substance use. An ultrasound-guided bone marrow aspiration and biopsy was performed 5 days later, to evaluate the pancytopenia. Per outside bone marrow report, his complete blood count (CBC) on the day of the bone marrow biopsy showed marked macrocytic anemia (RBC 2.23 M/cumm, Hgb 8.3 g/dL, hematocrit 24.3%, MCV 108.6 fL, MCH 37.2 pg, RDW 26.0%), moderate leukopenia (WBC 2.7 K/cumm), and moderate thrombocytopenia (45 K/cumm). His peripheral blood smear showed leukoerythroblastosis. His bone marrow aspirate and biopsy revealed a hypercellular bone marrow (85%) with marked erythroid hyperplasia and dysplasia, marked megakaryocytic hyperplasia, 13-14% blasts, and evidence of hemophagocytosis (slides not available for review). The findings raised concern for a COVID-19-induced hemophagocytic lymphohistiocytosis (HLH). In addition, a high-grade myelodysplastic syndrome could not be ruled out. The patient was thus referred to Harbor-UCLA Medical Center for further management after being tested negative for SARS-CoV-2 twice.

**Figure 1:**
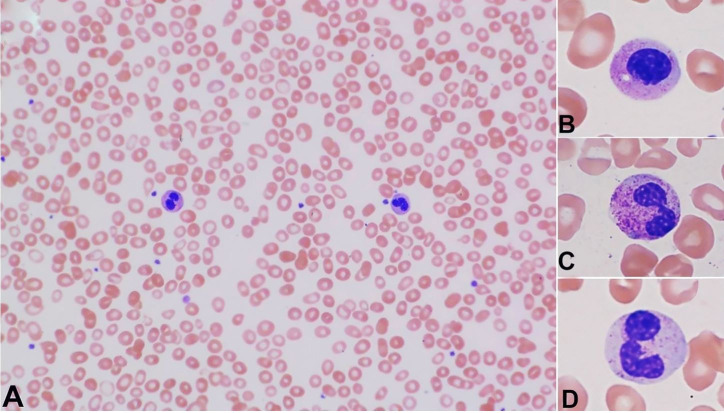
Photomicrograph of the peripheral blood smears showing occasional dysplastic neutrophils. **A** – The neutrophil in the left is dysplastic, with a bilobed nucleus (pseudo-Pelger-Huët anomaly). The neutrophil in the right is a normal-appearing band. A few teardrop cells are seen; **B** – A dysplastic neutrophil with a non-segmented nucleus; **C** – A dysplastic neutrophil with a bilobed nucleus; **D** – A dysplastic neutrophil with moderately hypogranular cytoplasm. [Wright-Giemsa stain, original magnification, × 1000 (A), × 1000 (B-D].

The patient presented at Harbor-UCLA Medical Center on the Day16 after initial symptoms, feeling well without fever/chills, weight loss, dizziness/lightheadedness, sweating, or pain. He had no history of exposure to pesticides, benzene, or other substances that may cause MDS. He denied recent alcohol, tobacco, or illicit drug use. The only medications he took were acetaminophen and ibuprofen in the previous month. He had no family history of hematologic malignancy. At presentation, the patient had an oral temperature of 36.9 °C, a heart rate of 92 beat/minute, blood pressure of 122/70 mmHg, and a respiratory rate of 18 breaths/min. The physical exam and chest X-ray were unremarkable. No splenomegaly was noted. A CBC showed moderate normochromic-macrocytic anemia (RBC 2.52 M/cumm, normal range 4.40-5.60 M/cumm; Hgb 9.4 g/dL, normal range 13.5 -16.5 g/dl; hematocrit 28.3%, normal range 40.0-49.0%; MCV 112.3 fL, normal range 82.0 -97.0 fL; MCH 37.4 pg, normal range 27.0-33.0 pg; MCHC 33.3 g/dL, normal range 32.0 – 35.0 g/dL; RDW 26.2%, normal range 12.0-15.0%), moderate leukopenia (WBC 2.6 K/cumm, normal range 4.5 -10.0 K/cumm; 100-cell manual differential: neutrophils 47%, lymphocytes 51%, monocyte 1%, and eosinophil 1%), and moderate thrombocytopenia (69 K/cumm, normal range 160-360 K/cumm) with increased large/giant platelets (MPV 12.9 fL, normal range 7.0-11.0 fL). Review of the peripheral blood smears revealed a leukoerythroblastic picture with occasional dysplastic neutrophils and a few teardrop cells (Figure 1).

Additional blood tests were performed, and the test results are summarized in Table 1.

**Table 1:** Blood test results at presentation to Harbor-UCLA Medical Center

Test	Result	Normal Range	Test	Result	Normal Range
Sodium	136	136-144 mmol/L	Haptoglobin	27	36-195 mg/dL
Potassium	4.1	3.6-5.1 mmol/L	Iron	150	95-182 mcg/dL
Chloride	102	101-111 mmol/L	Ferritin	565.3	23.9-336.2 ng/mL
CO_2_	27	22-32 mmol/L	TIBC	363	261-478 mcg/dL
BUN	13	8-20 mg/dL	Iron saturation	41	15-50%
Creatinine	0.91	0.64-1.27 mg/dL	Folate	9.9	≥ 5.9 ng/mL
Calcium	9.3	8.9-10.3 mg/dL	Vitamin B12	422	180-914 pg/mL
Total protein	7.0	6.4-8.3 g/dL	MMA	103	87-318 nmol/L
Albumin	4.5	3.5-4.8 g/dL	LDH	209	98-192 U/L
AP	60	38-126 U/L	Copper level	91	70-175 mcg/dL
AST	35	15-41 U/L	Triglyceride	101	≤ 149 mg/dL
ALT	69	10-40 U/L	Interleukin 2R	2067	532-1891 pg/mL
Total bilirubin	1.7	0.3-1.2 mg/dL	TSH	2.449	0.350-4.940 µIU/mL

ALT= Alanine transaminase; AP= Alkaline phosphatase; AST= Aspartate transaminase; BUN= Blood urea nitrogen; LDH= Lactate dehydrogenase; MMA= Methylmalonic Acid; TIBC= Total iron binding capacity; TSH= Thyroid-stimulating hormone

**Figure 2:**
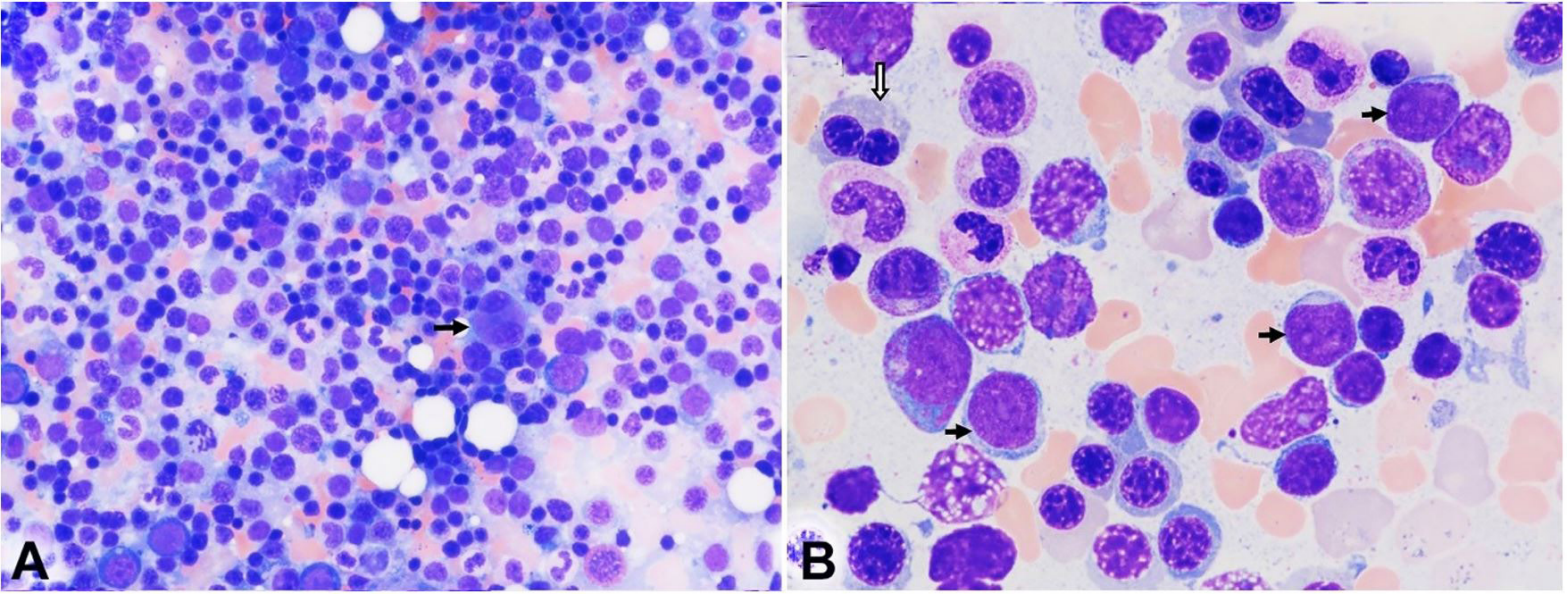
Photomicrographs of the bone marrow aspirate smears. **A** – Erythroid hyperplasia and some erythroblasts in apoptosis are evident. A dysplastic megakaryocyte is noted, with two separated round nuclei (arrow); **B** – Blasts (black arrows) are mildly increased. A binucleated erythroid precursor (block arrow) is seen. [Wright-Giemsa stain, original magnification, × 400 (A), × 1000 (B)].

**Figure 3:**
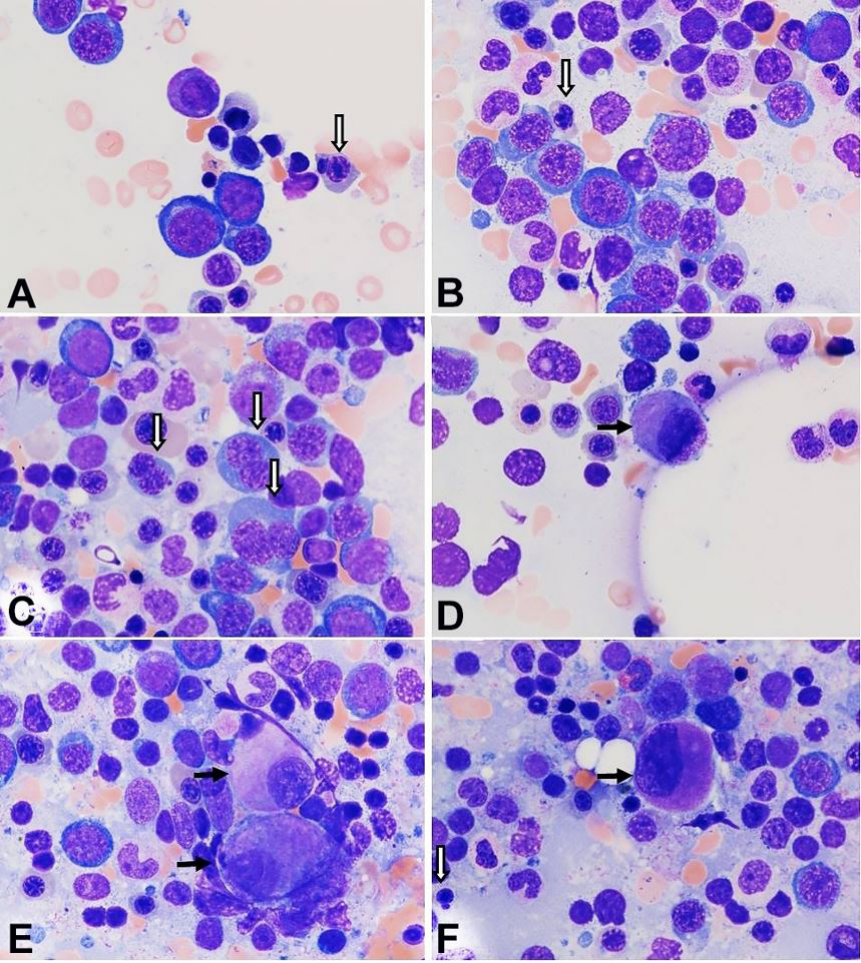
Photomicrograph of the bone marrow aspirate smears show mild dyserythropoiesis and some dysplastic megakaryocytes. There are a few erythroid precursor cells showing dysplastic features including abnormal nuclear budding (**A** and **B**), megaloblasts with “nuclei in salami-slice” (**B**), and binucleation (**C**). Micromegakaryocytes (**D**) and megakaryocytes with small non-lobated nuclei (**E**) are present. F. The megakaryocyte shows asynchronous nuclear-cytoplasmic maturation, with well granulated cytoplasm and a non-lobated immature nucleus. A dysplastic erythroid precursor is also seen. (Block arrows: dysplastic erythroid precursors; black arrow: dysplastic megakaryocytes; Wright-Giemsa stain, original magnification, × 1000).

Given the diagnostic uncertainty, a bone marrow aspiration and biopsy were repeated 20 days after the presentation at Harbor-UCLA Medical Center. The smears of bone marrow aspirate contained rare spicules showing marked erythroid hyperplasia and scattered blasts (Figure 2), without evidence of hemophagocytosis. A 500-nucleated cell count revealed the following differential: neutrophils and precursors 18.0%, erythroid precursors 58.6%, lymphocytes 7.6%, monocyte 0.4%, eosinophils 1.0%, plasma cells 1.4%, and blasts 13.0%. Dysplastic features were noted in a few erythroid precursors and some megakaryocytes (Figures 2 and 3).

**Figure 4:**
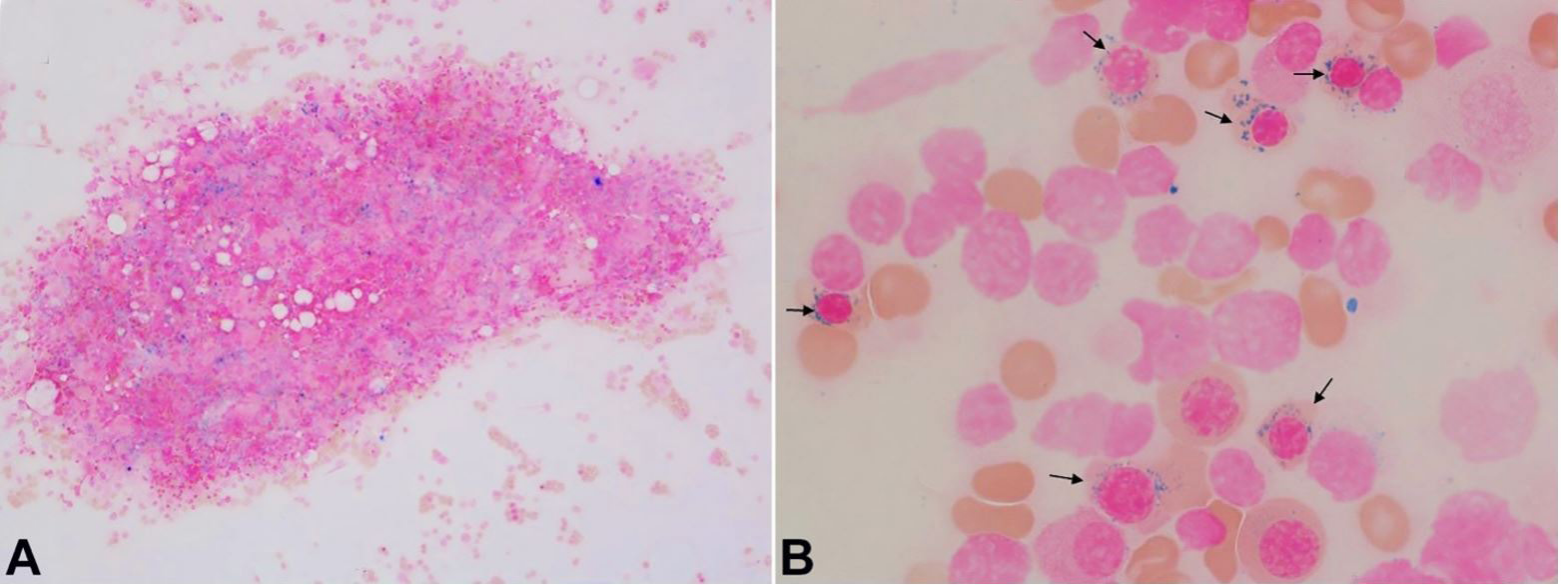
Photomicrograph of the iron stain of bone marrow aspirate showing adequate iron stores (**A**) with numerous ring sideroblasts (arrows, B). [Iron stain, original magnification, × 100 (A), × 1000 (**B**)]

Rare dysplastic neutrophils morphologically similar to those seen in the peripheral blood smear were also found. The Prussian blue stain performed on the bone marrow aspirate smear showed adequate stainable iron stores (Figure 4A) with many ring sideroblasts (53%, 100-cell count; Figure 4B).

**Figure 5:**
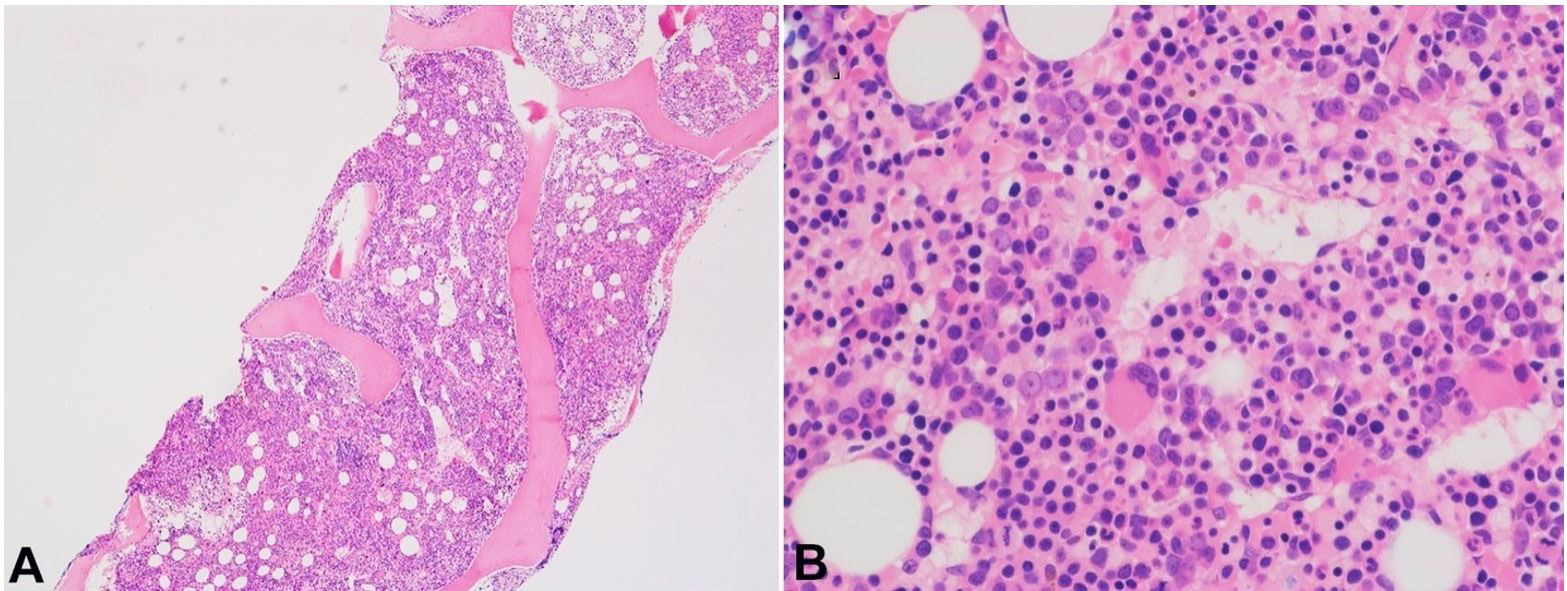
Photomicrograph of the bone marrow biopsy shows a hypercellular bone marrow (**A**) with trilineage hematopoiesis and erythroid proliferation (**B**). [H&E, original magnification, × 40 (A), × 400 (B)].

**Figure 6:**
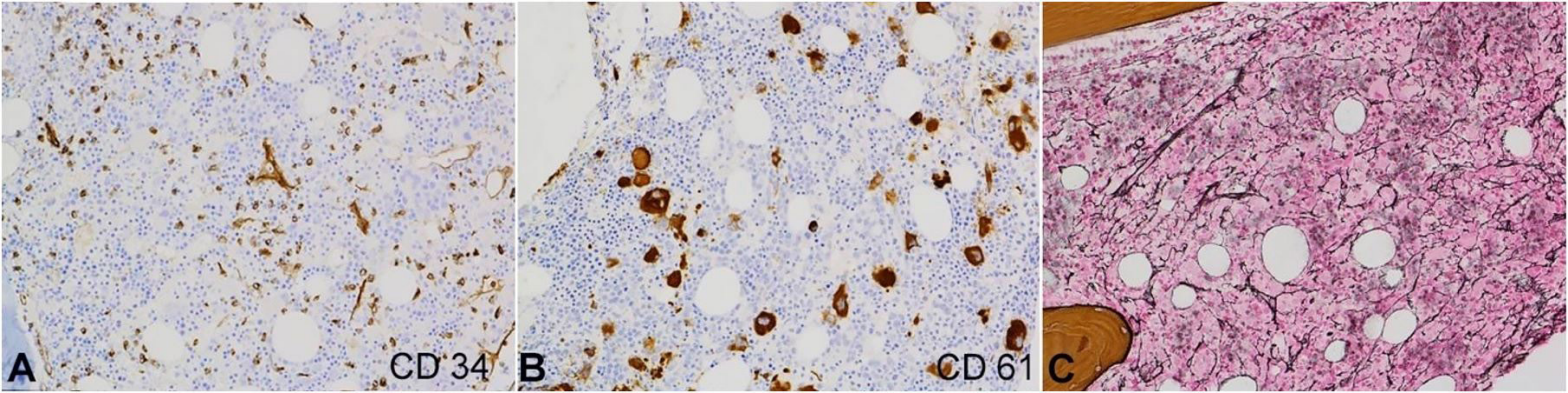
Photomicrograph of the bone marrow biopsy showing increased immature cells, most of which stain positive for CD34 (**A**). There are many megakaryocytes with various sizes, highlighted by CD61 stain, including several micromegakaryocytes (**B**). Reticulin stain of the marrow biopsy reveals a mild to moderate increase in reticulin fibers (**C**). (A-B, immunocytochemical stain, original magnification, × 200; C, reticulin stain, original magnification, × 200).

A flow cytometric study performed on the bone marrow aspirate showed the blasts expressed CD34, CD38, CD45 (weak), and CD117, and were negative for T-cell markers, B-cell markers, CD14 or CD64. The bone marrow biopsy sections revealed a markedly hypercellular bone marrow (90%) with marked erythroid proliferation (Figure 5), and increased immature cells, most of which stained positive for CD34 (Figure 6A). CD34-positive blasts represented approximately 13% of all bone marrow nucleated cells.

In addition, there are many megakaryocytes of various sizes, highlighted by CD61 stain (Figure 6B), including several micromegakaryocytes. The reticulin stain performed on the bone marrow biopsy showed mild to moderate myelofibrosis (MF 1-2/3, WHO 2017 semiquantitative grading of bone marrow fibrosis) (Figure 6C).

A diagnosis of myelodysplastic syndrome with excess blasts 2 (MDS-EB-2) was made. The bone marrow aspirate was sent to Quest Diagnostics Nichols Institute (San Juan Capistrano, CA) for cytogenetic and molecular tests. Chromosomal analysis revealed an abnormal clone with an isochromosome 14 resulting in trisomy 14 in 20/20 metaphase cells analyzed {46,XY,i(14)(q10)[20]}. LeukoVantage®, Myelodysplastic Syndrome (MDS) Panel using next generation sequencing demonstrated SF3B1 K700E mutation at 38.6% mutation frequency, ZRSR2 H191L variant at 49.2% variant frequency, and GATA2 G81R variant at 48.0% variant frequency, without alterations of *ASXL1, ATM, BCOR, CBL, CEBPA, CSF3R, CUX1, DNMT3A, ETNK1, ETV6, EZH2, FLT3, IDH1, IDH2, IKZF1, JAK2, KRAS, NF1, NPM1, NRAS, PHF6, PTEN, PTPN11, RUNX1, SETBP1, SRSF2, STAG2, STAT3, STK11, TET2, TP53, U2AF1,* and *WT1* genes. The patient was determined to have high risk disease according to the revised International Prognostic Scoring System (IPSS-R) and was referred for bone marrow transplant evaluation. While waiting for transplant evaluation, he was started on azacytidine therapy.

## DISCUSSION

These are the three “pillars” that support a diagnosis of MDS: 1. persistent and clinically unexplained cytopenia in at least one hematopoietic lineage (required); 2. significant morphologic dysplasia in one or more hematopoietic lineages (with the exception of cases bearing certain qualifying cytogenetic aberrations); and 3. cytogenetic and/or molecular genetic evidence of clonal hematopoiesis (not required, but helpful).^4^ A diagnosis of MDS can be challenging as the morphologic dysplasia and cytopenia may be related to factors other than a clonal hematologic disorder. Cytopenia can be seen in many non-MDS neoplasms in addition to reactive conditions such as infections, inflammations, metabolic deficiencies, the effects of drugs and alcohol intake, hemolysis, and autoimmune diseases. Dysplasia itself, even if prominent, is not definitive evidence of a clonal process because it is frequently seen in patients with reactive secondary cytopenia.^5^ ^-^ ^8^ Several nutritional (such as folic acid, vitamin B12 and copper deficiency), toxic (such as heavy metals particularly arsenic, lead and zinc, drugs) and other factors (such as congenital disorders, infections, and autoimmune disorders) can cause myelodysplastic changes but not atypia.

After emerging in December 2019 in Wuhan, China, the coronavirus disease 2019 (COVID-19) has rapidly spread and evolved into a global pandemic. The disease is caused by severe acute respiratory syndrome coronavirus 2 (SARS-CoV-2), but the pathophysiology of this infection is not completely understood. The disease affects multiple organs including hematopoietic system. In the peripheral blood, both quantitative and qualitative abnormalities have been reported. CBC and peripheral blood smear findings commonly reported in COVID-19 patients include anemia, leukopenia, leukocytosis, neutropenia, neutrophilia, lymphopenia, monocytosis, monocytopenia, eosinopenia, thrombocytopenia, thrombocytosis, and leukoerythroblastic picture.^9^ ^-^ ^13^ Morphologic aberrations were reported mainly in leukocytes and platelets, including changes usually found in MDS such as dysplastic neutrophils with hypogranular cytoplasm and/or hyposegmented nucleus (acquired Pelger-Huët anomaly) as well as giant platelets.^12^ ^-^ ^15^ Taken together, COVID-19 and MDS may present with similar peripheral blood findings. Further studies are needed to understand if and how hematopoiesis is involved in COVID-19 pathogenesis, and if COVID-19 patients have a higher susceptibility for secondary hematologic diseases.

Prieto-Pérez et al.^16^ studied post-mortem bone marrow samples from 17 patients who died with a diagnosis of COVID-19, and in most cases found increased bone marrow cellularity, scattered macrophages with engulfed erythrocytes and erythroblasts resembling the changes observed in HLH, left-shifted myeloid hyperplasia, clusters of megakaryocytes, and interstitial aggregates of CD8 positive lymphocytes. Our patient might have had HLH when he was diagnosed with COVID-19 at the outside hospital, given the very high serum ferritin level and the hemophagocytosis in his bone marrow. However, at Harbor-UCLA Medical Center, he had only mild hyperferritinemia, without fulfilling other diagnostic criteria for HLH. It is likely that his HLH had been largely resolved, although the only treatments he received were acetaminophen and ibuprofen. In the study conducted by Prieto-Pérez et al.^16^, it was not mentioned if COVID-19 patients’ bone marrow can show myelodysplastic features, possibly because myelodysplasia is best evaluated on bone marrow aspirate smears which is unobtainable post-mortem.

The diagnosis of MDS may be particularly challenging in patients with COVID-19 in whom it is hard to determine whether the peripheral blood and bone marrow abnormalities are caused by COVID-19 or MDS. In difficult cases, clinical follow-up may be required for the differentiation. Due to the diagnostic uncertainty, the patient was referred to our hospital for further diagnosis and management.

At our hospital, the diagnosis of MDS was made based on the presence of persistent pancytopenia including macrocytic anemia (folate and vitamin B12 deficiency had been excluded), marked bone marrow hypercellularity, trilineage myelodysplasia, numerous ring sideroblasts, and 13% myeloblasts. The diagnosis was further supported by abnormal cytogenetic and molecular results.

Blasts are normally 1% to 5% of bone marrow cells and are usually not seen in peripheral blood. Although up to 2% blasts have been reported in the peripheral blood of COVID-19 patients,^13^ an increase in blasts in bone marrow in the context of morphologic dysplasia and prolonged cytopenia in the absence of growth factor therapy, as seen in the present case, is a very strong indicator of MDS. To the authors’ knowledge, increase in bone marrow blasts (≥5%) has not been reported in reactive conditions such as acute viral infection in the literature.

A cytogenetic abnormality characteristic of MDS confirms the diagnosis, but cytogenetic abnormalities are only seen in about 50% of MDS cases. According to the 2017 WHO classification, MDS-associated cytogenetic abnormalities that are sufficient to confirm a diagnosis of MDS in a cytopenic patient include loss of chromosome 7 or del(7q), del(5q), isochromosome 17q or t(17p), loss of chromosome 13 or del(13q), del(11q), del(12p) or t(12p), del(9q), idic(X)(q13), t(11;16)(q23.3;p13.3), t(3;21)(q26.2;q22.1), t(1;3)(p36.3;q21.2), t(2;11)(p21;q23.3), inv(3)(q21.3;q26.2)/t(3;3)(q21.3;q26.2), and t(6;9)(p23;q34.1).^3^ On the other hand, some common cytogenetic abnormalities seen in MDS, i.e., gain of chromosome 8, del(20q), and loss of Y chromosome, can be seen in normal aged persons or in individuals with non-MDS caused cytopenia,^17^ ^-^ ^19^ and are not considered definitive evidence of MDS. In our patient, chromosomal analysis revealed an abnormal clone with an isochromosome 14 resulting in trisomy 14 in 20/20 metaphase cells analyzed {46,XY,i(14)(q10)[20]}. Trisomy 14/14q is extremely rare in myeloid neoplasms, mainly seen in MDS and chronic myelomonocytic leukemia (CMML), and less frequently observed in AML or other myelodysplastic/myeloproliferative neoplasms (MDS/MPN).^20^ Associated with intermediate to good prognosis, this abnormality occurs as an isolated change in less than 0.5% of patients with MDS and is typically found in older, male patients with and without excess blasts.^20^ In the largest series of myeloid neoplasms with isolated trisomy 14 (n=16) published so far, 5/16 patients showed an isolated isochromosome 14q [i.e., i(14)(q10)] resulting in trisomy 14q.^21^ We report, for the first time, this cytogenetic aberrancy as isolated change in a young MDS patient.

Acquired somatic gene mutations are seen in the vast majority of MDS patients at diagnosis, with several commonly recurring mutations such as *SF3B1, TET2, ASXL1, SRSF2, DNMT3A, RUNX1, U2AF1, TP53, EZH2, STAG2, IDH1/IDH2, CBL, NRAS*, and *BCOR*. Many myeloid-associated somatic mutations have been identified in healthy older individuals without MDS,^22^ ^-^ ^23^ known as “clonal hematopoiesis of indeterminate potential” (CHIP). For these reasons, genetic mutations alone cannot be used to support clonality or make a diagnosis of MDS, even with cytopenia, in the 2017 WHO classification.^3^ Mutation of SF3B1 was detected in our patient, which is closely associated with MDS with ring sideroblasts and MDS/MPN with ring sideroblasts and thrombocytosis. Given the young age of our patient, the detected SF3B1 mutation is very likely disease-related rather than CHIP-associated. Therefore, in our case, the presence of numerous ring sideroblasts and the detection of SF3B1 mutation further support the diagnosis of MDS.

## CONCLUSION

Peripheral blood of COVID-19 patients may show cytopenia and dysplastic neutrophils, which are features commonly seen in patients with MDS, making accurate diagnosis of MDS more challenging during the COVID-19 pandemic. Clinicians should be aware that although commonly seen in elderly patients, MDS can occur in young individuals. Besides cytopenia and myelodysplasia, macrocytic anemia (without vitamine B12 or folate deficiency) and ring sideroblasts increase the suspicion of MDS. If present, excess blasts (≥5%) and/or MDS-qualifying cytogenetic abnormalities support a diagnosis of MDS. Genetic mutations alone cannot be used to support clonality or make a diagnosis of MDS, but could be helpful in some situations. In difficult cases, a close follow-up of the patient may be required for further diagnostic clarification.

## References

[1] Ma X, Does M, Raza A, Mayne ST (2007). Myelodysplastic syndromes: incidence and survival in the United States. Cancer.

[2] Rollison DE, Howlader N, Smith MT (2008). Epidemiology of myelodysplastic syndromes and chronic myeloproliferative disorders in the United States, 2001-2004, using data from the NAACCR and SEER programs. Blood.

[3] Hasserjian RP, Orazi A, Brunning RD, Swerdlow SH, Campo E, Harris NL (2017). Myelodysplastic syndromes: overview.. WHO classification of tumours of haematopoietic and lymphoid tissues..

[4] Hasserjian RP (2019). Myelodysplastic syndrome updated. Pathobiology.

[5] Bain BJ (1996). The bone marrow aspirate of healthy subjects. Br J Haematol.

[6] Parmentier S, Schetelig J, Lorenz K (2012). Assessment of dysplastic hematopoiesis: lessons from healthy bone marrow donors. Haematologica.

[7] Steensma DP (2012). Dysplasia has a differential diagnosis: distinguishing genuine myelodysplastic syndromes (MDS) from mimics, imitators, copycats and impostors. Curr Hematol Malig Rep.

[8] Della Porta MG, Travaglino E, Boveri E (2015). Minimal morphological criteria for defining bone marrow dysplasia: a basis for clinical implementation of WHO classification of myelodysplastic syndromes. Leukemia.

[9] Huang C, Wang Y, Li X (2020). Clinical features of patients infected with 2019 novel coronavirus in Wuhan, China. Lancet.

[10] Chen N, Zhou M, Dong X (2020). Epidemiological and clinical characteristics of 99 cases of 2019 novel coronavirus pneumonia in Wuhan, China: a descriptive study. Lancet.

[11] Rodriguez-Morales AJ, Cardona-Ospina JA, Gutiérrez-Ocampo E (2020). Clinical, laboratory and imaging features of COVID-19: a systematic review and meta-analysis. Travel Med Infect Dis.

[12] Ahnach M, Ousti F, Nejjari S, Houssaini MS, Dini N (2020). Peripheral blood smear findings in COVID-19. Turk J Haematol.

[13] Lüke F, Orsó E, Kirsten J (2020). Coronavirus disease 2019 induces multi-lineage, morphologic changes in peripheral blood cells. EJHaem.

[14] Nazarullah A, Liang C, Villarreal A, Higgins RA, Mais DD (2020). Peripheral blood examination findings in SARS-CoV-2 infection. Am J Clin Pathol.

[15] Zini G, Bellesi S, Ramundo F, d’Onofrio G (2020). Morphological anomalies of circulating blood cells in COVID-19. Am J Hematol.

[16] Prieto-Pérez L, Fortes J, Soto C (2020). Histiocytic hyperplasia with hemophagocytosis and acute alveolar damage in COVID-19 infection. Mod Pathol.

[17] Gupta V, Brooker C, Tooze JA (2006). Clinical relevance of cytogenetic abnormalities at diagnosis of acquired aplastic anaemia in adults. Br J Haematol.

[18] Soupir CP, Vergilio JA, Kelly E, Dal Cin P, Kuter D, Hasserjian RP (2009). Identification of del(20q) in a subset of patients diagnosed with idiopathic thrombocytopenic purpura. Br J Haematol.

[19] Jacobs KB, Yeager M, Zhou W (2012). Detectable clonal mosaicism and its relationship to aging and cancer. Nat Genet.

[20] Bacher U, Schanz J, Braulke F, Haase D (2015). Rare cytogenetic abnormalities in myelodysplastic syndromes. Mediterr J Hematol Infect Dis.

[21] Cui W, Bueso-Ramos CE, Yin CC (2010). Trisomy 14 as a sole chromosome abnormality is associated with older age, a heterogenous group of myeloid neoplasms with dysplasia, and a wide spectrum of disease progression. J Biomed Biotechnol.

[22] Busque L, Patel JP, Figueroa ME (2012). Recurrent somatic TET2 mutations in normal elderly individuals with clonal hematopoiesis. Nat Genet.

[23] Laurie CC, Laurie CA, Rice K (2012). Detectable clonal mosaicism from birth to old age and its relationship to cancer. Nat Genet.

